# Relationship between clinical manifestations and serological profile in patients affected by Systemic Lupus Erythematosus

**DOI:** 10.3389/fimmu.2024.1390642

**Published:** 2024-08-16

**Authors:** Stefania Nicola, Richard Borrelli, Federica Corradi, Luca Lo Sardo, Iuliana Badiu, Alessandra Romito, Nicolò Rashidy, Anna Quinternetto, Marina Mazzola, Federico Meli, Elena Saracco, Ilaria Vitali, Domenico Cosseddu, Luisa Brussino

**Affiliations:** ^1^ Department of Medical Science, Allergy and Clinical Immunology Unit, Mauriziano Hospital, University of Turin, Turin, Italy; ^2^ Department of Laboratory Medicine, Mauriziano Hospital, Turin, Italy

**Keywords:** lupus, SLE, autoantibodies, serological profile, immunology

## Abstract

**Background:**

Systemic lupus erythematosus (SLE) is a chronic autoimmune disorder characterized by a variety of both signs and symptoms; it mainly affects women of childbearing age, with an estimated prevalence of 24/100,000 people in Europe and North America. SLE is often described as an antibodies-driven disease as its clinical manifestations are usually associated with the presence or the absence of specific antibodies.

**Objectives:**

To evaluate clinical manifestations in patients with SLE and to assess the relationship with the presence of specific antibodies by using real-world data.

**Methods:**

A retrospective study was performed; the 2019 EULAR/ACR Classification Criteria for Systemic Lupus Erythematosus were used to classify patients with SLE. Data concerning serological profiles (which included Antinuclear antibodies – ANA, anti dsDNA, anti-Ro/SS-A, anti-La/SS-B, anti-Smith) were gathered along with medical records of clinical manifestations. Complement levels were also tested for possible clinical correlations. χ² or Fisher’s exact tests were utilized to establish associations between autoantibodies and symptoms. The odds ratios (OR) and their 95% confidence intervals (CI) were computed. No correction was made for multiple testing; only a p-value 0.01 ≤ was considered significant.

**Results:**

One-hundred and twenty-seven patients (n=127, mean age 53.43 ± 14.02) were enrolled in this study. Anti-dsDNA antibodies were found to be statistically significant for both malar rash and proteinuria; anti-Ro/SSA antibodies showed an association with photosensitivity and pericarditis; furthermore, a strong association was found between anti-Ro antibodies and proteinuria, but only if anti-dsDNA antibodies were present as well. Patients who tested positive for anti-La/SSB antibodies correlated with a threefold increase in the risk of developing pericarditis. Lastly, anti-Smith appeared to be associated with NPSLE as well as an increased risk for both autoimmune hemolytic anemia and thrombocytopenia.

**Conclusions:**

In our study, many associations confirmed those found in previous studies; however, new relationships between antibodies and clinical manifestations were found thus indicating the need for additional evaluations to assess these correlations further.

## Introduction

### Background

Systemic lupus erythematosus (SLE) is a chronic autoimmune disease that causes inflammation and can damage almost any organ and system in the body. It can present with a wide range of symptoms, and the presence of certain antibodies may be related to specific manifestations (1).

To date, the etiology of the disease has not been thoroughly assessed; however, multifactorial participation has been established as it incorporates epigenetic, genetic, ecological, and environmental components (2–4).

### Epidemiology

Following the estimates provided by the worldwide epidemiological community, the proportion of females to males is nine to one (F: M 9:1), with the age range of 15 to 44 years old being the highest (5). It has been estimated that the incidence of SLE is 5.14 (1.4; 15.13) per 100 thousand person-years; however, this number can vary greatly depending on the region of interest, ranging from 1.18 (0.16; 3.68) per 100 thousand person-years in central Asia to 13.74 (3.2; 31.82) per 100 thousand person-years in central Europe. Regarding ethnicity, Systemic Lupus Erythematosus appears to be significantly more frequent in African American individuals when compared to Caucasians (6–8).

### Pathogenesis and autoantibodies

SLE is a classic example of an autoimmune disease that is caused by the formation of immunocomplexes. An abnormal immune response, along with a change in the process of clearing nucleic acids, leads to the loss of self-tolerance and the production of autoantibodies, which ultimately form pathogenic immunocomplexes. These immune complexes carrying self-DNA and RNA stimulate the production of excessive amounts of type I interferon (IFN) by plasmacytoid dendritic cells via Toll-like receptors seven and nine (TLR7 and TLR9) and are responsible for complement activation leading to damages in the various organs. Type I IFN also affects B-cell activity as it promotes enhanced survival and activation, which includes differentiation and class-switch recombination and can result in the formation of autoantibodies (9–11).

Anti-nuclear antibody (ANA) positivity has gained greater importance in classifying SLE in recent literature. ANA is believed to contribute to disease progression by impacting the immune system and organs such as the brain, kidneys, and skin, and it comprises several autoantibodies, including anti-nucleosome, anti-dsDNA, anti-histone, anti-Smith (Sm), anti-Ribonucleic Protein Antigen (RNP), anti-Ro, and anti-La antibodies, each with specific immunofluorescence pattern (12, 13).

While anti-Sm and anti-dsDNA antibodies are specific to SLE, the others are also found in other autoimmune diseases (14). Moreover, the level of anti-dsDNA antibody is a crucial autoantibody biomarker for the SLE Disease Activity Index (SLEDAI) (15).

### Diagnosis and classification

The most recent classification criteria were edited in 2019 by the “European League Against Rheumatism/American College of Rheumatology” (ACR/EULAR) to include patients in an early stage of disease (16); in fact, their sensitivity is 93% (95% CI, 0.83-0.98), which is higher than the 2012 Systemic Lupus International Collaborating Clinics (SLICC) criteria (83%, 95% CI, 0.72- 0.91) (17). However, the newest criteria did not improve specificity, showing a comparable percentage than the previous ones (75%, 95% CI, 0.61-0.85 for the EULAR/ACR VS 73%, 95% CI, 0.59-0.83 for SLICC).

Unlike the previous ones, the presence of an entry criterion (the presence of ANA) is now mandatory for the classification into studies; this element defines a noticeable difference with cohorts from previous studies (14); furthermore, items are now divided into clinical and immunological domains; patients are considered affected if the score is at least 10.

### Treatment

The recent update of the recommendations (2023 ACR/EULAR Recommendations on the management of Systemic Lupus Erythematosus) changed the therapeutic approaches for patients with SLE. Hydroxychloroquine (HCQ) is the mainstay of treatment and should be used at a dosage of no more than 5 mg/kg. Prednisone or equivalent is also often required for inducing remission and for chronic maintenance, with a dosage lower than 7.5 mg daily and, wherever feasible, should be stopped if not required. In addition, an immunosuppressant can be considered in individuals who are refractory to HCQ therapy (with or without glucocorticoids or too high dosages). Unlike the previous recommendations, biological therapies like Belimumab (anti-Blys) and Anifrolumab (anti-IFN Receptor 1) can be administered in mild form of disease as they proved to be both safe and effective for patients (18).

Furthermore, Voclosporin, a novel calcineurin inhibitor, was the last medication added to the list of LN in combination with mycophenolate (19). Lastly, in cases of severe disease, like those involving CNS or renal involvement, Cyclophosphamide (CYC) or Rituximab (RTX) should be used (18).

## Materials and methods

### Study design and population

This retrospective monocentric cohort observational study enrolled outpatients with a confirmed diagnosis of SLE according to the 2019 ACR/EULAR classification criteria with systemic lupus erythematosus (16).

To ensure the quality of the data, patients with inadequate or incomplete medical documentation were excluded from the study. We also applied a range of exclusion criteria, including active neoplasia, diagnosis of primary or secondary immunodeficiency, and substance abuse.

### Objectives

The main objective is to assess the correlation between the autoantibody subtypes and the clinical manifestations, in order to verify any possible differences with the previous studies and assist clinicians in predicting possible outcomes of the disease. Possible disease outcomes can be predicted by considering clinical manifestations and serological profiles, which could help establish specific treatment plans for SLE patients.

### Data collection

Data concerning age, sex, smoking habits, ongoing and previous treatments, as well as comorbidities were collected.

### Serological evaluation

Data on the serological profiles of patients were collected. These data include antinuclear antibodies (ANA, HEp-2 IFA by Euroimmun S.r.L, Italy), anti-double-stranded DNA antibodies (anti-dsDNA, evaluated on fluorescent enzyme immunoassays - FEIAs - by ThermoFisher, Waltham, MA and IFA on Chrithidia luciliae -Euroimmun S.r.L. for positive findings in order to confirm the result), anti-Ro/SS-A antibodies (divided, where available, into 52 kDa and 60 kDa, evaluated on fluorescent enzyme immunoassays - FEIAs by ThermoFisher), anti-La/SS-B and anti-Smith (anti- Sm, FEIAs byThermoFisher) antibodies (20–22).

Complement levels, namely C3 and C4, were also evaluated with blood tests to determine their trend and possible correlation with clinical manifestations.

### Systemic and organ involvement

Organ involvement was assessed by using the definitions of 2019 ACR/EULAR for Systemic Lupus Erythematosus (supplementary material, Table 1 of the classification criteria) and the Safety of Estrogen in Lupus National Assessment - Systemic Lupus Erythematosus Disease Activity Index (SELENA-SLEDAI) (23).

The presence of hematological manifestations (namely thrombocytopenia, lymphopenia, and hemolytic anemia) was evaluated with seriated blood tests with a focus on platelets, hemoglobin, WBC, and lymphocytes, haptoglobin, Lactate Dehydrogenase (LDH), direct and indirect bilirubin levels; direct Coombs tests were also performed. Joint manifestations (including arthralgia, Jaccoud’s arthritis, non-deforming non-erosive arthritis, and erosive arthritis) were assessed by the presence of either two or more joints with pain and signs of inflammation (tenderness, swelling or effusion) or ultrasound (US) of the joints with Power Doppler (PWD).

Cutaneous manifestations were defined as acute lupus, discoid lupus alopecia, presence of oral ulcers or malar rash; renal involvement was evaluated by the presence of glomerulonephritis (established with a kidney biopsy) or the presence of proteinuria >0.5 g/L in a 24-hour urine collection.

Neuropsychiatric involvement was assessed in the presence of encephalitis, psychosis, epilepsy, or depression, which could not be related to other plausible causes; pericarditis or pleuritis with or without pericardial or pleural effusion were investigated as per 2019 ACR/EULAR definitions on Systemic Lupus Erythematosus (16, 24, 25).

### Statistical analysis

The information that was gathered was analyzed with the help of STATA SE 18.0 (1985-2023 StataCorp LLC, College Station, Texas, United States of America).

For the purpose of establishing connections between autoantibodies and symptoms, either the χ2 or Fisher’s exact tests were applied. Both the odds ratios (OR) and the confidence intervals (CI) for each of them were computed 95% There were no adjustments made to account for multiple testing; as such, the only p value that was considered significant was 0.01 or less.

### Ethical approval

All of the subjects gave their consent after being fully informed. All of the procedures were carried out in accordance with the pertinent rules, in accordance with the Declaration of Helsinki from 1964, and in accordance with the regulations that were imposed by the legislative body. This study was approved by the local Ethics Committee (Comitato Etico Territoriale interaziendale AOU Città della Salute e della Scienza di Torino, study #0116953).

## Results

One-hundred and twenty-seven patients (n=127) were enrolled in this study as per inclusion criteria; demographics and records can be found in **Tables 1**, **2a**, **2b**, **3**.

**Table 1:** Demographic characteristics.

Sex	Numbers of patients	Frequency rate
Male	13	10.24
Female	114	89.76
Total	127	100.00
**Mean age:** 53.43 (SD 14.02)		**F:M ratio:** 8.77:1

**Table 2:** Distribution of autoantibodies.

a) ANA Pattern (ICAP)	Numbers of patients	Frequency rate	Other ANA Patterns
AC-1	84	66.14	AC-1 only
AC-4	36*	28,35	*36 out of 36 also showed AC-1
AC-8	3*	2,36	*7 out 8 also showed AC-1
AC-14	2*	1,57	*1 out of 2 also showed AC-8
AC-21	2*	1,57	*2 out of 2 also showed AC-1
ANA: AntiNuclear Antibody, AC pattern: AntiCell pattern			
b) ENA	Number of patients	Frequency rate	
Anti dsDNA	77	60,63	
Anti-Ro/SSA	49	38,58	
Anti-La/SSB	18	14,17	
Anti-Sm	16	12,60	

Anti dsDNA, anti-double-stranded DNA antibody; anti-SSA, Anti-Sjogren’s Syndrome A; anti-SSB, anti Sjogren’s Syndrome B; anti Sm, anti Smith.

**Table 3:** Distribution of clinical manifestations.

Clinical manifestations	Numbers of patients	Frequency rate
Malar rash	67	52,76
Photosensitivity	79	62,20
Proteinuria	33	25,98
Pericarditis	29	22,83
Pleuritis	24	18,90
NPSLE	14	11,02
AIHA	13	10,24
Thrombocytopenia	42	33,07
Leukopenia	24	18,90

NPSLE, NeuroPsychiatric Systemic Lupus Erythematosus; AIHA, Autoimmune hemolytic anemia.

As shown in the tables, the demographic characteristics of the enrolled patients are comparable to those found in literature.

Alas, different patterns of Antinuclear antibodies (ANA) failed to demonstrate a significant association with specific clinical manifestations As concerns the other types of autoantibodies, the results are summarized and shown in **Table 4**.

**Table 4:** Relationship between clinical manifestations and antibodies that reached statistical significance.

Antibody	Clinical manifestation	OR (95% CI)	p-value	no of patients with the clinical manifestation (%)
**Anti dsDNA**	Malar rash	2.35 (1.13; 4.87)	**0.010**	**67 (52.75)**
** **	Proteinuria	3.13 (1.24; 7.92)	**0.008**	**33 (25.98)**
**Anti Ro/SSA**	Photosensitivity	2.64 (1.20; 5.82)	**0.007**	**79 (62.20)**
** **	Pericarditis	2.92 (1.25; 6.85)	**0.006**	**29 (22.83)**
**Anti Ro/SSA AND Anti dsDNA**	Proteinuria	7.13 (1.39; 36.66)	**0.010**	**14 (11.02)**
**Anti La/SSB**	Pericarditis	3.56 (1.25; 10.16)	**0.009**	**28 (22.05)**
**Anti Sm**	NPSLE	5.15 (1.46; 18.12)	**0.005**	**14 (11.02)**
** **	AIHA	5.85 (1.63; 21.02)	**0.003**	**13 (10.24)**
** **	Thrombocytopenia	5.68 (1.82; 17.67)	**<0.001**	**42 (33.07)**

Anti dsDNA, anti-double-stranded DNA antibody; anti-SSA, Anti-Sjogren's Syndrome A; anti-SSB, anti Sjogren's Syndrome B; anti-Sm, anti Smith.

Values in bold are statistically significant.

Anti dsDNA antibodies were found to be statistically significant for both malar rash (OR 2.35, CI 95% 1.13; 4.87, p=0.01) and proteinuria (OR 3.13, CI 95% 1.24; 7.92, p=0.008).

Anti-Ro/SSA antibodies showed an association with photosensitivity (OR 2.64, CI 95% 1.20; 5.82, p=0.007) and pericarditis (OR 2.92, CI 95% 1.25; 6.85, p=0.006). A strong association was found between anti Ro antibodies and proteinuria, but only in the cohort of patients who also tested positive for anti dsDNA; in fact, this association appeared to be related with a sevenfold increased risk for patients in case of double positivity (OR 7.13, CI 95% 1.39, 36.66, p=0.010).

A different scenario was ascertained in patients who tested positive for anti La/SSB antibodies; in our cohort these antibodies were found to be related with a threefold increase in the risk of pericarditis (OR 3.56, CI 95% 1.25, 10.16, p=0.009). Lastly, anti-Smith antibodies were evaluated; its presence appeared to positively correlate with NPSLE (OR 5.15, CI 95% 1.46, 18.12, p=0.005); as for the hematological manifestations, anti-Smith antibodies increased the risk for both autoimmune hemolytic anemia (OR 5.85, CI 95% 1.63, 21.02, p= 0.003) and thrombocytopenia (OR 5.68, CI 95% 1.82, 17.67, p< 0.001).

Lastly, the relationship between clinical manifestations and complement levels were tested; as showed in **Table 5**, reduced levels of complement were statistically significant for the development of proteinuria (OR 3.40, CI 95% 1.39, 8.31, p=0.007).

**Table 5:** Relationship between clinical manifestations and complement levels.

Complement	Clinical manifestation	OR (95% CI)	p-value	no of patients with the clinical manifestation (%)
**C3, C4**	Proteinuria	3.40 (1.39; 8.31)	0.007	**33 (25.98)**

C3, Complement factor 3; C4, Complement factor 4.

Values in bold are statistically significant.

## Discussion

SLE is a disease characterized by a combination of clinical manifestations that can widely vary among affected patients; as such, it might be helpful to use real-world data to assess possible correlations between antibodies and clinical manifestations.

The population described in our study is comparable to the cohorts described in the literature for both sex distribution and age (26, 27).

As mentioned in the results, diverse patterns of ANA were not statistically significant for different clinical manifestations among patients with SLE. This result might be explained by the usage of a set of criteria which require ANA to be present as the main inclusion condition; unlike studies from other countries who used ACR criteria (in which differences could be observed due to the heterogeneity of the cohort), our patients were similar and all of them were positive for the presence of ANA. Moreover, the great majority of the patients showed an AC-1 pattern, thus reducing the chance to observe any statistically significant difference.

Patients with anti-dsDNA antibodies saw a statistically significant association with the presence of proteinuria and malar rash, which appear to agree with previous studies (14, 28, 29); however, unlike other evaluations, pleuritis, alopecia and lymphopenia did not reach a significant correlation (30).

Proteinuria was also observed in patients with a double positivity for both anti Ro/SS-A antibodies and anti dsDNA as their copresence appeared to be significant for a sevenfold risk of its development. Additionally, anti-Ro/SSA antibodies correlated with increased photosensitivity for patients who tested positive, which has been assessed in previous studies as well (31); a correlation with pericarditis was also observed in these patients, unlike the results found in other reports as they failed to show a correlation with anti-SSA antibodies (32, 33).

Our work managed to confirm the relationship between anti-La/SSB antibodies and pericarditis, which was observed in previous studies (21, 34) but did not show a significant correlation with renal involvement nor with other manifestations of SLE.

This element might be explained by the difference between the cohorts; in the study of Novak and colleagues, patients only needed to fulfill the American College of Rheumatology (ACR) definition of SLE, which does not require an entry criterion (namely, the presence of ANA); moreover, they needed to have a diagnosis of SLE before 18 years of age. Childhood- onset SLE (cSLE) usually shows, in fact, a more severe combination of constitutional signs and symptoms when compared to patients with a later onset (35).

Lastly, it has already been established that patients from different ethnicities might develop non-identical manifestations of SLE as patients of Latin ethnicity tend to manifest a renal involvement more frequently than Caucasian patients (36).

In our cohort of patients, anti-Smith antibodies were highly associated with hematological pictures, namely lymphopenia and AIHA as well as NPSLE, in agreement with previous reports (37, 38).

Interestingly, we didn’t find any significant association between anti-Smith antibodies and the presence of proteinuria, renal involvement, lymphopenia, or cardiac involvement, which was observed in other studies (36, 39). Similarly to before, this difference might be explained by taking into account the epidemiological elements on which these studies have been conceived.

The main work on this topic comes from the Genetic Profile Predicting the Phenotype study (PROFILE), which is a well-characterized multi-ethnic cohort of SLE patients constituted in 1998; not only was the study based on the 1997 American College of Rheumatology (ACR) revised criteria, but it also enrolled patients who were either Latin-American, Hispanic or African- American. As such, different clinical manifestations are to be expected as this cohort appears to be rather specific and poorly comparable to the one analyzed in this study.

We also checked for possible correlations between low levels of complement and clinical manifestations in our sample; in agreement with previous studies, our work confirmed that patients with reduced C3 and/or C4 levels might develop proteinuria (40, 41).

In conclusion, relationships between clinical manifestations and antibodies have been widely assessed in the past but different definitions of disease and new laboratory methods changed the cohorts of patients in which we can verify such associations. Among these, many relationships have confirmed previous reports, whilst others have diverged or described new possible relationships which might benefit from further investigations.

## Limitations

The main limitation of this study is its retrospective design with data collected from medical history and recorded from a single center. Another limitation which might be taken into consideration is the high prevalence of Caucasian patients in our population, unrepresentative of other ethnic groups.

## Data Availability

The raw data supporting the conclusions of this article will be made available by the authors, without undue reservation.
